# Told You So

**Published:** 1896-08

**Authors:** 


"TOLD YOU SO." "JUST AS I EXPECTED."—There are not in the English language perhaps, three words of more significance than those above; nor is there anything that can be said that is of more satisfaction to the say-er, or more aggravating to the said-to. (It is the remark the old lady made when informed that the cow had swallowed the grindstone). It may be taken home to one's self as evidence of a superior discernment on his part, and it makes the other fellow feel small; he stands convicted of a mistake,—an error of judgment or a foolish thing.

Now, didn't we tell you so about the homeopaths not accepting the offer of the State Medical Association to play second fiddle in the game of medical legislation? Our remark was about to the intent and purpose as follows: "What assurance is there that they (the homeopaths) will be satisfied with this proportion of representation,—four to our six? Having obtained that amount of concession,—secure now of recognition, will they not grow more bold and presumptuous, and demand complete and full fellowship on boards and in consultation room?

Just as we predicted. Indeed, is it not drawing it pretty fine to recognize homeopaths as a "school of medicine"—our equals in everything, invite them to participate in efforts at legislation, to sit on boards with us, and then deny to individual members consultation with them, on pain of expulsion? No wonder they cannot see it; the line is too fine. They know they hold the vantage ground, and can dictate terms. It is the whole hog or none; and they are right. Having defeated us in every effort at legislation, they know, and the Association confesses that legislation without their consent, is not possible; and now that the Association has humbled itself to them, they spurn the advance, and say in effect, "Gentlemen, the entire swine if you please." It would seem that being forewarned was not to be fore armed in this instance; for it was well known to the medical profession that the homeopaths had declared they would oppose any bill coming from that source. We told you so.

The question now is, what is going to be done about it? Will the Association allow the Homeopaths to dictate the terms upon which they will condescend to affiliate on medical boards? Or, will the Association heed our admonition to let them alone? It is an embarrassing position to be placed in, for which the Association has no one to blame but themselves; a situation which every friend of that body must regret.

